# Smartphone dependency, digital amnesia, and somatic symptoms among nursing students: the challenge of artificial intelligence

**DOI:** 10.1186/s12912-025-03228-0

**Published:** 2025-05-26

**Authors:** Lamiaa Abd El Hakeem Ali, Maha Ibrahim El Bayoumy Ali, Soheir Mohammed Ahmed Ali, Abdulqadir J. Nashwan

**Affiliations:** 1 Psychiatric Mental Health Nursing, Faculty of Nursing, Cairo University, Giza, Egypt; 2 Community Health Nursing Department, Faculty of Nursing, Cairo University, Giza, Egypt; 3 Nursing Administration Department, Faculty of Nursing, Cairo University, Giza, Egypt; 4 Nursing Department, Hamad Medical Corporation, Doha, Qatar

**Keywords:** Smartphone dependency, Digital amnesia, Somatic symptoms, Nursing students, Artificial intelligence

## Abstract

**Background:**

As we advance into the digital era, the latest challenge in smartphone technology is integrating artificial intelligence applications, which have recently been incorporated into learning environments. However, the intensified engagement with these devices has raised concerns about potential issues among nursing students. This study aimed to assess the relationship between Smartphone Dependency, Digital Amnesia, and Somatic Symptoms among nursing students who face Artificial Intelligence challenges.

**Method:**

A cross-sectional study was conducted in three public and three governmental universities in Egypt from July 1st to July 31st. A convenient sample of 495 nursing students participated in the study. Four instruments were used for data collection: The artificial intelligence usage questionnaire, the digital amnesia scale, the test of mobile phone dependence (TMD), and the somatic symptom scale-88 (SSS-88).

**Results:**

The findings indicated a prevalence of low to moderate smartphone dependency, moderate digital amnesia, and a very high level of somatic symptoms among nursing students. Besides, AI usage and smartphone dependency had a statistically significant relation. As well, there was a statistically significant relation between smartphone dependency and both digital amnesia and somatic symptoms, *P*-value > 0.001.

**Conclusion:**

The findings revealed that the role of AI presented challenges among nursing students and indicated a statistically significant relation between AI usage and smartphone dependency among nursing students (*P* < 0.000). Additionally, there was a statistically significant relation between smartphone dependency and both digital amnesia and somatic symptoms (*P* < 0.001, *P* < 0.000), respectively.

**Recommendations:**

The findings of this study highlight the crucial role of key stakeholders, particularly nursing educators, in implementing targeted educational interventions. These include integrating digital well-being into nursing curricula and promoting practices such as digital breaks/digital detox periods in the classroom. Such strategies aim to encourage mindful smartphone use and help prevent comorbidities like dependency, digital amnesia, and somatic symptoms.

**Clinical trial number:**

Not applicable.

**Supplementary Information:**

The online version contains supplementary material available at 10.1186/s12912-025-03228-0.

## Introduction

The digital technology revolutions of recent decades have significantly enhanced comfort and convenience in our daily lives, with a profound impact on how we think, learn, behave, and remember [1]. Terms such as “digitalization” and “artificial intelligence” have evolved into prominent concepts in contemporary discourse, frequently highlighted in media and shaping our societal narratives [2]. The COVID-19 pandemic further accelerated this shift, making online learning and teaching methods supported by digital tools like smartphones and artificial intelligence a common practice. The utilization of digital devices effectively and efficiently has become the demand of the hour, notably during this pandemic [3]. The rapid rise in smartphone usage, with an estimated 6.1 billion users globally by 2020, reflects a significant societal shift toward mobile technology [4].

Technology facilitates student-centered learning by empowering students to participate more actively in their education. This approach enhances the learning experience to address individual needs [5]. Technology increasingly supports students by improving the availability of educational resources and expanding access to information, offering instruments that extend learning outside the academic setting [6]. These tools deliver active and multisensory paths to provide content. Many students rely heavily on their smartphones, which have placed several electronic devices like watches, cameras, calculators, radios, and tape recorders, contributing to what some authors describe as making students brighter [1]. Additionally, the utilization of this technology is growing within the medical training field. Utilizing disruptive technologies in health care or medical programs facilitates teaching and prepares students for a highly integrated workplace with advanced technology [7].

Digitalization offers substantial benefits, including enhanced innovation, efficiency, sustainability, and competitiveness. However, transitioning to a culture prepared for the digital age presents challenges [8]. While digital technology provides significant benefits, its misuse can lead to various health hazards [9]. Dirin, Alamäki, & Suomala [2], noted that global life expectancy has significantly increased compared to the last century. Even though these advances, the advancement of digital technological revolutions have also been associated with negative consequences for persons and society. Song & Park [10] observed that although a learning environment enhanced by technology can foster achievement in the classroom, it may also foster dependency on the smart device.

Dependency on smartphones is described as symptoms expressed through behavior linked with pleasant and often seductive [11]. Ahn, Jun, & Kim [12] describe dependency on smartphones as not merely about the frequency of smartphone use involves a deeper psychological relationship between the user and their device. Lobo et al. [13] explained dependency as characterized by repeated checking of messages, the development of tolerance evidenced by prolonged smartphone utilization, feelings of fear without using the device, and deterioration in function attributed to the device’s disruption of various life activities and social interactions.

Silva & Nanamura [6] asserted that excessive dependence on smartphones could adversely impact an individual’s memory, potentially leading to digital amnesia, whereby individuals came to perceive these devices as substitutes for their memory. Lodha addressed the phenomenon of digital amnesia as a rising threat to human memory due to overuse and dependence on technology devices. It is also referred to as the atrophy of memory induced by the technology [14]. Digital Amnesia is the encounter of missing information that humans trust digital devices will store and recall.

Smartphone use and related thoughts can affect the accuracy of memory recall, showing that they contribute to increased cognitive loading, disrupting memory processes [15]. The widespread adoption and integration of smart devices into daily life have reduced the need for individuals to remember personal details, such as loved ones’ phone numbers or their schedules for the upcoming day. With smartphones functioning as personal assistants, individuals can easily access this information without relying on memory [16].

Digital devices are entrusted with valuable and confidential information about the person, involving contacts and images, and also provide users with access to a vast repository of knowledge, available at any time and from any location. As youth increasingly use digital devices for online studies, this growing dependency can harm personal development. Prolonged use may contribute to physical inactivity and the development of somatic symptoms [3]. According to Savarimuthu & Subramanian [17], smartphone dependency can replace personal memories with those stored on devices. Beyond memory issues, digital amnesia is associated with behavioral matters such as distractibility, impulsivity, and increased aggressiveness, which can extend to impact physical health and lead to somatic symptoms.

Somatic symptoms do not indicate a special disorder but reflect an underlying pathological process. These physical symptoms cause significant distress and/or dysfunction, yet they cannot be adequately explained by conventional medicine. Physiological factors are increasingly recognized as contributing to somatic symptoms [18]. These symptoms, often linked to brain functions, may reveal underlying emotional conflicts. Common examples include fatigue, headache, chest pain, dizziness, and abdominal discomfort [19]. While the psychological impact of dependency on smartphones, such as digital amnesia, has received considerable attention, it is equally important to recognize the physical manifestations, such as somatic symptoms, that may accompany such a behavioral pattern [20–21].

Cerutti et al. [22] emphasized that the development trends and societal needs suggest that dependency on digital devices will increase significantly, particularly as progressions in artificial intelligence (AI) better occupy consumers emotionally and anticipate their needs. Furthermore, Small et al. [9] stated the risks and effects of widespread technology use include impaired emotional and social intelligence, addiction, socialization, atrophy of brain development, and sleep disturbance. Dienlin & Johannes [23] added, digital technology does not simply happen to students; rather, students actively engage with it, often with a high level of competence.

Nursing students, in particular, are vulnerable due to the intense demands and pressure of their educational programs. This stress can lead to frequent smartphone use for social interaction and relaxation, increasing the risk of dependency [13]. Smartphone dependency among nursing students may be linked to impaired in the function of cognition, low self-esteem, sleep disturbance, reduced physical activities, pain, headache, and diminished cognitive control [24]. As smartphones become indispensable, students may feel incomplete without them [12, 10].

A comprehensive review of 266 studies involving over 22,500 undergraduate nursing students offers valuable insights into the current use of digital educational tools in the education of nursing curricula. The findings underline the potential of these tools to enhance learning experiences and outcomes for nursing students significantly [25]. So, assessing smartphone dependency, digital amnesia, and somatic symptoms in a representative sample of nursing students from both governmental and private Faculties of nursing in Egypt requires significant attention since it has not been addressed worldwide.

### Significance of the study

Forster’s 1909 short story, The Machine Stops, presents a futuristic scenario where a mysterious machine controls everything, from supplying food to information technologies, creating a world where face-to-face communication is absolute, and society entirely depends on this machine. This narrative parallels today’s digital landscape, where digital media dominate our interaction and information consumption. This story highlights how such dependency can lead to societal collapses when the controlling force fails [26].

This concept of dependency extends to contemporary issues such as smartphone addiction, which has been extensively studied and documented [4, 27]. The rapid advancement of artificial intelligence (AI) has only intensified this dependency, shaping how we interact with technology in our daily lives [27]. This evolution presents new challenges, especially for nursing students who increasingly rely on smartphones for their educational and professional needs [13]. So, the current over-reliance on technology raises significant concerns about its potential impacts on societal structure and individual well-being. However, excessive dependence on smartphone technology can impair long-term memory [28]. Furthermore, prolonged smartphone use impacts mental and physical health, leading to somatic symptoms [29, 30]. Moreover, in a clinical environment, excessive use of smartphones may be a major distraction, resulting in negligence and adverse effects on patient care and safety [31].

Finally, the intersection of smartphone dependency, digital amnesia, and somatic symptoms highlights a critical issue; as AI continues to shape our digital landscape, it is crucial to assess these variables and the associated challenges that impact nursing students. Moreover, to date, no previous studies in Egypt have explored the combined relationship between these variables. Based on that, we want to better understand how smartphones affect the way that nursing students recall and utilize the information today, for which level they are dependent on the smartphone, and if it can develop any somatic symptoms to formulate a picture regarding the significant relation between all of these factors and artificial intelligence contribution. Highlighting the significant gap that this study aims to address.

### Aim of the study

This study aims to assess the relationship between smartphone dependency, digital amnesia, and somatic symptoms among nursing students, on the challenges posed by artificial intelligence.

## Subjects and methods

### Research questions

The research questions that this study seeks to answer are:

1. What are the levels of smartphone dependency, digital amnesia, and somatic symptoms of nursing students?
2. What is the correlation between smartphone dependency, digital amnesia, somatic symptoms, and AI usage among nursing students?
3. What impact does artificial intelligence usage have on smartphone dependency, digital amnesia, and somatic symptoms among nursing students?

### Research design

This study used a cross-sectional correlational design to examine the correlation between smartphone dependency, digital amnesia, and somatic symptoms.

### Setting

This study was conducted through online applications such as WhatsApp and Facebook. A link to a Google form containing the study questionnaires was sent to nursing students at six different faculties in Egypt: three private institutions (Modern University for Technology and Information (MTI), Misr University for Science and Technology (MUST), and 6 October University) and three governmental institutions (Cairo University, Ain Shams University, and Helwan University). These organizations were selected because they represent the largest number of nursing students in Egypt moreover, attract students from both urban and rural areas, offering a more representative view of nursing students across different contexts.

### Study sample

This study selected a convenience sample of 495 nursing students using smartphones for at least one year and agreed to participate based on the Yamane [32] formula. Students who had never used a smartphone or were unfamiliar with AI were excluded from the study because the study specifically aimed to assess the relationship between smartphone dependency with digital amnesia, and somatic symptoms. Including students without adequate usage would have limited the validity of the finding.

The formula is: numerator $$\:\frac{{z}^{2}*p*(1-p)/{e}^{2}}{1+\:\frac{{z}^{2}*p*(1-p)}{{e}^{2}*N}}$$

n: Sample size $\:398$, N: Total population 62.614, E: Acceptable margin of error, a significance level of 0.05, a Confidence Level of 95% confidence interval. According to Central Agency for Public Mobilization and Statistics Egypt (2023) the study’s target population comprised approximately 62.614 nursing students enrolled in selected Egyptian Universities during the 2023–2024 academic year. These universities included governmental institutions such as Cairo University, Ain Shams University, and Helwan University, as well as private universities including Misr University for Science and Technology (MUST), 6th October, and the British University in Egypt. Nursing students from all academic years who met the inclusion criteria and agreed to participate were selected to ensure broad participation. A non-random convenience sampling strategy was used due to its practicality and efficiency, especially under time constraints. Efforts were made to minimize selection bias by distributing the questionnaire widely across different academic levels within selected faculties. The initial formula calculation estimated 398, a slightly larger number to account for the exclusion of nursing students who did not use smartphones or were unfamiliar with AI.

The sample included 415 nursing students from governmental institutions and 80 nursing students from private universities, followed by 230 nursing students from Cairo University, 125 nursing students from Ain Shams University, and 60 nursing students from Helwan University, in addition to 22 nursing students from Misr University for Science and Technology (MUST),14 nursing students from 6th October, and 44 nursing students from The British University in Egypt(MTI).

### Tools of data collection

**The first tool**,** the artificial intelligence usage questionnaire**, was developed by researchers (Supplementary file 1) and consists of two parts. The first part includes sociodemographic data: It was used to collect data from nursing students and encompasses items such as age, gender, marital status, residence, name of university, and educational level. **The second part includes** a description of the pattern of artificial intelligence usage. For example, Which AI app or tools do you mostly use? How often do you use artificial intelligence apps? And for what, using an AI app? The reliability of the developed questionnaire was confirmed by a statistician using Cronbach’s alpha of 0.79, indicating an acceptable level of internal consistency with satisfactory validity.

**The second tool**,** the digital amnesia scale**,** a structured scale** originally developed by Kaspersky Lab [33] was adapted to measure the phenomena of digital amnesia. The scale consists of 13 items. Questions 1 through 8 are scored as if the student exhibits signs of digital amnesia and 0 otherwise. For questions 9 through 13, each item is scored as follows: points for agree, 1 point for neutral, and 0 points for disagree. The total score is categorized as follows: Low (0–6), Moderate (7–12), High (13–18). Data interpretation is based on percentage distribution. The reliability of the translated Arabic version of the scale was confirmed by a statistician using Cronbach’s alpha of 0.80, indicating an acceptable level of internal consistency with satisfactory validity.

**The third tool**,** The Test of Mobile Phone Dependence (TMD)**, a structured scale developed by Chóliz [34] consists of 22 items designed to assess University students’ usage and dependency on smart devices. The first 10 items are answered on a Likert scale ranging from 0 (never)to 4 (frequently), while the remaining 12 items use a Likert scale ranging from 0 (completely disagree) to 4 (completely agree). The total TMD score ranges from 0 to 88, with scores indicating Low (0–44), Moderate (45–66), and High (67–88) dependency. The test demonstrated good reliability with a Cronbach’s alpha = 0.94 for the original version and 0.91 for the translated Arabic version.

**The fourth tool**,** the Somatic Symptom Scale-8 (SSS-8)**, is a brief self-report questionnaire developed by Gierk et al. [35] to assess somatic symptom burden. It measures the perceived burden of common somatic symptoms with respondents rating themselves on a 5-point Likert scale ranging from 0 (not at all), 1 (a little bit), 2 (somewhat), 3 (quite a bit), to 4 (very much). The SSS-8 severity categories are calculated by percentile ranks: no to minimal (0–3 points), low (4–7 points), medium (8–11 points), high (12–15 points), and very high (16–32 points). The SSS-8 demonstrated excellent item characteristics and good reliability, with a Cronbach alpha of 0.81 for the original version and 0.84 for the Arabic translation.

### Procedure

The researchers reached out to the students via WhatsApp and Facebook to fulfill the aim of the current study. An electronic version of the questionnaire (Google Form) was designed and distributed for ease of access and response, practicality, and efficiency, especially given time constraints and the need to reach many participants across different academic levels. The questionnaire was distributed through faculty WhatsApp groups and online learning platforms restricted to current students. Participants were provided with detailed information about the study, including its nature and the option to participate, with confidentiality assured. Only students who agreed to participate completed the study questionnaires via Google Forms, available for one month from July 1st to July 31st, 2023/2024 academic year. Students were assured of the confidentiality of their responses. Furthermore, reminders were sent during the data collection to encourage participation and improve response rates. The study utilized tested structured questionnaires to ensure reliability and validity. The researchers employed translation and back-translation procedures for the Digital Amnesia Scale, the Test of Mobile Phone Dependence (TMD), and the Somatic Symptom Scale-8 (SSS-8) to accommodate Arabic-speaking students. The original English versions were first translated into Arabic and then reviewed by other bilingual experts for accuracy. Other bilingual experts back-translated the resulting Arabic versions into English to ensure fidelity. A statistician confirmed the reliability of the Arabic versions. These tested structured questionnaires were selected based on their relevance to the study aims and their established use in related research. Each tool measures an interrelated construct central to our research questions, and they provide a comprehensive framework for understanding the relationships among digital amnesia, smartphone dependence, and somatic symptoms among nursing students.

### Ethical consideration

The study was conducted following the Declaration of Helsinki ethical guidelines. Primary approval was obtained from the Scientific Research Ethics Committee, Faculty of Nursing, Cairo University, Egypt **(**No, PHDIRB2019041701**)**, to conduct this study. Participation in the study was voluntary and based on obtaining the participants’ informed consent through submitting the completed study tools. The ethical considerations include explaining the purpose and nature of the study to keep the information confidential and ensure that others would not access it. Electronic data were collected anonymously and stored securely, with access restricted to the research team. The study did not pose any risk to the participants.

### Statistical design

SPSS version 22.0 was used for data entry and statistical analysis. Descriptive statistics were applied, including numbers, percent, mean, and standard deviation. The Pearson correlation coefficient was used to study correlation, and linear regression analysis was conducted to assess the impact of variables. ANOVA was used to examine the relationship between sociodemographic data and study variables. Chi-square tests and t-tests were performed to identify significant differences between variables. Internal consistency for all three translated and developed tools was computed using Cronbach’s alpha, with an alpha value greater than 0.70 considered acceptable.

## Results

Table 1 reveals that 59.8% of the studied sample were female, and 48.1% were aged between 21 and 23 years, with a Mean age of 22.4. Concerning marital status, 81.2% of the participants were single, and 63.2% resided in urban areas. In addition, 42.6% of the studied sample were in their internship year. Furthermore, 83.9% attended governmental universities, with 46.5% affiliated with Cairo University.

**Table 1:** Personal characteristics of nursing students (*n* = 495)

Personal characteristics	No.	%
**Gender**		
Female	296	59.8
Male	199	40.2
**Age**		
18-20Yrs	113	22.8
21-23Yrs	238	48.1
24-28Yrs	144	29.1
**Mean 22.4**		
**Marital status**		
Single	402	81.2
Married	90	18.2
Divorced	3	0.6
**Residence**		
Rural	182	36.8
Urban	313	63.2
**Educational Level**		
Internship	211	42.6
First	39	7.9
Third	93	18.8
Second	63	12.7
Forth	89	18.0

Table 2 shows that 71% of the studied sample used AI for entertainment and education. In addition, 55% of nursing students use Chat GPT as their preferred AI application, followed by 12.1% using Google Assistant/Translate/Lens. Regarding AI usage, 45.3% of government students and 48.8% of private students usually use AI, with a statistically significant association between the two types of universities (*P* < 0.002).

**Table 2:** Artificial intelligence usage of nursing students (*n* = 495)

Artificial intelligence usage	No.	%
**Use of AI for**		
Entertaining	26	5.3
Education	116	23.4
Both	353	71.3
**Most AI apps used**		
ChatGPT	272	55
Google Assistant/ translate/ lens	60	12.1
Smart camera	36	7.2
Grammarly	30	6.1
True caller	30	6
Face recognition	25	5.1
Smart scan	24	4.8
Share it	9	1.8
GPS	5	1
Siri	4	0.8
**Governmental AI use**		
Sometimes	158	38.1
Usually	188	45.3
Rarely	69	16.6
**Private AI use**		
Sometimes	37	46.2
Usually	39	48.8
Rarely	4	5.0
**Differences in AI usage**	**X**^**2**^ = 7.4	*P* = 0.020*

Table 3 indicates that 64.9% of the studied sample had a moderate level of digital amnesia, while only 4.8% exhibited a high level. Additionally, 46.3% of the participants demonstrated low smart device dependency, whereas 42.0% showed moderate dependency.

**Table 3:** Level of digital amnesia and dependency on smart devices of nursing students (*n* = 495)

Levels	Digital amnesia		Smartphone dependency	
	No.	%	No.	%
Low	150	30.2	229	46.3
Moderate	322	64.9	208	42.0
High	24	4.8	58	11.7

Table 4 shows that 47.5% of the studied sample had very high levels of somatic symptoms, while only 4.8% had no to minimal symptoms.

**Table 4:** Level of somatic symptoms of nursing students (*n* = 495)

Levels	Somatic symptoms	
	No.	%
No to minimal	24	4.8
Low	34	6.9
Medium	96	19.4
High	106	21.4
Very high	235	47.5

Table 5 clarifies a statistically significant relation between AI usage and smart device dependency among nursing students (*P* < 0.000). Additionally, there is a statistically significant relation between smartphone dependency and both digital amnesia and somatic symptoms (*P* < 0.001, *P* < 0.000), respectively.

**Table 5:** Relation between study variables among nursing students (*n* = 495)

Variables	AI usage		Digital amnesia		Smartphone dependency		Somatic symptoms	
	*R*	*P*	*r*	*P*	*R*	*p*	*R*	*p*
AI usage	1							
Digital amnesia	-0.08	0.06	1					
Smartphone Dependency	0.16	0.000*	0.14	0.0001*	1			
Somatic symptoms	0.008	0.85	0.03	0.39	0.38	0.000*	1	

Table 6 presents a significant positive impact of AI usage on smartphone dependency (B = 3.6, t = 3.7, *p* = 0.000). In contrast, the impact of AI on both digital amnesia and somatic symptoms is insignificant (B = -0.34, t = -1.88, *p* = 0.06), (B = 0.06, t = 0.18, *p* = 0.85) respectively.

**Table 6:** Impact of AI usage on study variables among nursing students (*n* = 495)

Variables	Impact of AI usage			
	B	s.e	T	*P*
Digital amnesia	-0.34	0.18	-1.88	0.06
Smartphone Dependency	3.6	0.99	3.7	0.000*
Somatic symptoms	0.06	0.37	0.18	0.85

*Significant p-value < 0.05

Table 7 illustrates a positive statistically significant relation between digital amnesia and both type of university and educational level (*P* < 0.049* & *P* < 0.001*) respectively. Furthermore, there was a positive statistically significant relation between dependency on smart devices and (age, type of university, name of university & educational level) (*P* < 0.012*, *P* < 0.000*, *P* < 0.007*, & *P* < 0.000*) respectively. Additionally, there was a positive statistically significant relation between somatic symptoms and (gender, age, type of university, name of university &educational level) (*P* < 0.000*, *P* < 0.017*, *P* < 0.000*, *P* < 0.000* & *P* < 0.000*) respectively.

**Table 7:** Relation between study variables and personal characteristics of nursing students (*n* = 495)

Personal characteristics	Digital amnesia		Smartphone dependency		Somatic symptoms	
	Mean	Sd	Mean	Sd	Mean	Sd
**Gender**						
Female	8.06	2.76	46.39	16.35	15.66	5.57
Male	7.60	3.15	46.94	15.67	12.93	5.98
ANOVA + p	2.96	0.08	0.142	0.707	26.883	0.000*
**Age**						
18-20Yrs	7.81	2.90	42.83	15.69	13.25	6.21
21-23Yrs	8.03	3.04	47.17	16.85	14.75	5.76
24-28Yrs	7.69	2.77	48.64	14.57	15.29	5.71
ANOVA + p	0.63	0.53	4.482	0.012*	4.097	0.017*
**Marital status**						
Single	7.86	3.01	46.21	16.40	14.39	6.03
Married	7.89	2.55	48.26	14.67	15.42	5.13
Divorced	9.67	1.15	50.00	8.66	12.00	6.93
ANOVA + p	0.56	0.56	0.660	0.517	1.413	0.244
**Residence**						
Rural	7.74	2.73	47.40	15.76	14.66	6.02
Urban	7.96	3.04	46.15	16.25	14.51	5.82
ANOVA + p	0.68	0.40	0.701	0.403	0.073	0.787
**Type of University**						
Governmental	7.77	2.87	45.46	16.39	14.06	5.94
Private	8.44	3.18	52.58	12.76	17.21	4.81
ANOVA + p	3.50	0.049*	13.500	0.000*	20.033	0.000*
**Name of University**						
Cairo	7.67	3.18	45.56	17.67	13.77	6.02
Ain Shams	7.92	2.39	44.83	14.17	13.90	5.78
Helwan	7.83	2.51	46.37	15.78	15.48	5.86
6 of October	8.71	3.10	49.00	13.67	17.71	3.36
MUST	9.18	3.51	56.45	15.28	19.09	3.89
MTI	7.98	3.02	51.77	10.77	16.11	5.34
ANOVA + p	1.34	0.24	3.199	0.007*	5.729	0.000*
**Educational Level**						
Internship	7.55	3.01	49.85	13.73	15.38	5.63
First	8.05	3.26	39.10	18.29	11.44	6.68
Third	8.02	2.67	44.69	16.21	13.13	5.38
Second	7.41	2.87	44.17	16.74	15.10	6.29
Forth	8.78	2.73	45.93	17.98	15.13	5.74
ANOVA + p	3.32	0.01*	5.184	0.000*	5.692	0.000*

## Discussion

In recent years, artificial intelligence (AI) has become an integral part of daily life, embedded in various devices [3]. Among these devices, smartphones have gained popularity, especially among students. Smartphones serve as tools for teaching, communication, and entertainment [1]. However, this reliance on smartphones for information retrieval may hinder cognitive processes related to memory retention, leading to digital amnesia. Furthermore, continuous engagement with smartphones has been associated with physical manifestations of emotional distress, including headache and body pain, contributing to the rise of somatic symptoms [36].

Firstly, it was observed that nearly half of the students (46.3%) demonstrated low dependency, while a smaller proportion (42.0%) exhibited moderate dependency. In the same stream, Rakesh et al. [37] reported similar findings among nursing students, with more than half indicating a moderate level of smartphone dependence. Additionally, Farghaly, Dator, & Sankarapandian [11] found that most nursing students tended to exhibit moderate to low levels of dependency. Furthermore, Turan et al. [38] in line with these findings that students’ dependency levels were moderate, indicating that the increasing internet frequency suggests unregulated and potentially problematic usage.

Moreover, Kalal et al. [39] found that 61.95% of nursing students exhibited mild smartphone addiction, while 38.1% demonstrated a moderate level. The researcher concluded that, despite the high proportion of nursing students with mild smartphone addiction, it was associated with an increased risk of poor sleep quality and diminished academic performance. These findings reinforced general observations of parallel distribution between low and moderate levels of smartphone dependency among nursing students; they highlight the risk of excessive smartphone use without adequate caution, especially with AI usage. Our findings revealed that nearly half of the nursing students in governmental (45.3%) and private (48.8%) universities usually used AI during the daytime, with 71% using it for entertainment and education.

Regarding digital amnesia, our results revealed that despite most nursing students having low and moderate levels of dependency on smartphones, more than half of students (64.9%) experienced moderate digital amnesia, highlighting the potential consequences of this issue. Similarly, Savarimuthu & Subramanian [17] found that digital amnesia was more prevalent among youth aged 18 to 25 years and concluded their findings by raising awareness about the risks and challenges of digital amnesia on cognitive, affect, and behavioral aspects among them. In agreement, Kim [40] underlined the link between smartphone dependence and memory loss among nursing students, emphasizing the need for targeted interventions within the educational environment. Based on the results of this study indicates that dependency on smartphones influences the performance of nurses in the clinical workplace, and if not well controlled, it can potentially lead to some problems such as physical, psychological, and social for all nurses, patients, and society. Consequently, the relevant authorities must examine developing rules and regulations for using smartphones in training and classroom workplaces.

In contrast, George & DeCristofaro [41] found that smartphone apps promote nursing students’ long-term retention of knowledge and provide immediate access to current clinical resources, highlighting the importance of evaluating the effectiveness of these tools on long-term nursing education. From the researcher’s point of view, smartphone apps can aid in developing nursing students’ skills, improving their knowledge, and facilitating communication with nurses in different countries for experience exchange. However, students must also be aware of the potential adverse effects and how to manage them. Understanding the digital impact on information processing is crucial to comprehending the cognitive consequences and challenges of reliance on digital technologies for memory-related tasks.

The level of somatic symptoms revealed that nearly half (47.5%) of nursing students experienced very high levels of these symptoms. Our finding is consistent with those of Backåberg, Rask, Brunt & Gummesson [42] who reported significant nursing students experiencing musculoskeletal symptoms during nursing education. These symptoms are common among nursing student and may impact their overall well-being and participation in physical activities. Similarly, an integrative review by Firmino et al. [43] highlighted the prevalence of somatic disorders among nursing students, emphasizing the pain and discomfort they often experience.

Moreover, a scoping review by Sperling et al. [44] on the prevalence of somatic symptoms among medical students, which included twenty-nine articles, found that somatic symptoms reached 80.1%. These symptoms were overwhelmingly correlated with mental ill-health, leading the authors to recommend future research exploring interventions to reduce physical symptom burden in medical students. From our perspectives, it is well understood that the nursing curriculum is inherently stressful, requiring students to not only master complex medical knowledge but also continuously update their skills to keep pace with evolving technology. However, the high prevalence of severe somatic symptoms suggested the education process itself may be overwhelming and contribute to diminished capacity to cope with stress.

Secondly, the results of this study illuminated a statistically significant relation between smartphone dependency and combined AI usage, digital amnesia, and somatic symptoms among nursing students. This aligns with the findings of Farghaly, Dator, & Sankarapandian [11], who showed a significant correlation between smart device addiction and the use of artificial intelligence among nursing students. Additionally, the study’s findings agree with the study conducted by Toda, Ezoe, Mure &Takeshita [45] showing that smartphone dependence positively correlated with somatic symptoms.

Also, a study by Musa, Mukhtaruddin & Bakkara [46], indicated that excessive use of digital devices for information storage and recall potentially led to digital amnesia. The study highlighted that the digital tools used by participants revealed lower memory retention and shallower information processing compared to those employing traditional memory strategies. We consider that students fortunate enough to own a smartphone integrate these devices deeply into their daily lives. They use smartphones not only for communication and entertainment but also for learning and capturing moments to share on social media, extending to calculators, calendars, alarms, digital notes, password storage, and navigation. This dependence on technology for cognitive tasks may inadvertently impair natural abilities such as memory retention and problem-solving, as students rely more on external aids than internal cognitive processes.

Our research showed no statistically significant correlation between digital amnesia and somatic symptoms (*p* = 0.39). This is in contrast with a study by Robert & Kadhiravan [36] who illuminated that somatic symptoms among youth were significantly impacted by digital amnesia and sleep disorders. Explained the significant difference in somatic symptoms among youth based on their demographic data such as gender, type of family, and area of place of residence. This discrepancy may be attributed to the widespread impact COVID-19 radically altering students’ daily routines over the last two years, leading to reliance on digital gadgets and greater digital dependency during virtual learning.

Thirdly, our findings underscore the significant impact of AI usage on smartphone dependency, demonstrating a statistically significant correlation between digital amnesia and somatic symptoms. Zhang et al. [30] in the same line found that frequent smartphone usage is contributing to a growing mobile phone addiction, which is harming sleep quality and memory decline. Revealing the potential impact of mobile phone addiction on daily memory function and sleep quality. It is also found that this relationship is universal across different geographical and cultural backgrounds, providing theoretical and empirical evidence for related fields concerning mobile phone addiction’s effects. Atalla & Mohamed [48] highlighted the critical role of ethical awareness in shaping positive attitudes toward AI, promoting innovative practices in nursing, and ultimately supporting nurses’ overall well-being.

Finally, the research results asserted a statistically significant positive relationship between somatic symptoms and variables such as gender, age, type of university, and educational level. As well as a study by Robert & Kadhiravan [36] found a smartphone dependency showed a statistically significant positive relationship with age, type of university, &and educational level. In agreement with the findings of Bhanderi, Pandya, & Sharma [47] the prevalence of smartphone use and addiction among adolescents aged 16–19 was reported to be 37%. This addiction was associated with factors such as age, area of residence, place of education, duration of smartphone use, daily hours of use, and education. Additionally, the study found a significant relationship between digital amnesia and the type of university, suggesting that the nature of the institution, whether governmental or private, affects how students use the smartphone, memorize information, and affect their physical health.

### Limitation

The first limitation of the study used the cut-off values of the digital amnesia score scale determined by the statistician rather than relying on established norms due to the absence of a validated score for digital amnesia. Although these recommended cut-off values are widely used in research studies across different populations, the validity of this scale was subsequently assessed. The second study focused on Egyptian nursing students, but technology use among students can vary significantly across countries and cultures. So, conducting a comparative cross-sectional survey across different cultural contexts may provide valuable insights and enhance the generalizability of findings. The third cross-sectional studies capture data at a single point in time, which limits the ability to establish causal relationships between excessive AI usage on smartphones, dependency, digital amnesia, and somatic symptoms. This design does not account for changes over time or the direction of influence, making it difficult to determine whether AI usage leads to dependency on smartphones, digital amnesia, and somatic symptoms. The fourth using online platforms for data collection, may introduce selection bias by potentially excluding students with limited or no internet access, which could influence the generalizability of the findings.

## Conclusion and recommendation

In conclusion of this study, the findings revealed that the role of AI presented challenges among nursing students, with a low to moderate prevalence of smartphone dependency, moderate digital amnesia, and a very high level of somatic symptoms. Based on these findings, it is recommended to develop educational interventions through initiatives and programs to guide nursing students in effectively using smartphones, such as guidelines in classrooms, stress management workshops. Educators should also be involved in designing programs that promote health and prevent comorbidities related to excessive smartphone use. Encouraging the practice of digital breaks/digital detox periods in the classroom and digital well-being workshops to help nursing students improve cognitive functioning, emotional well-being, and behavioral aspects. Further research is recommended to explore the effectiveness of these interventions across a broader range of age groups and diverse cultural backgrounds beyond university students, and include participants from different cultural backgrounds. This will help to generalize findings. This will improve the generalizability and applicability of findings. Longitudinal studies may be needed to explore the impact of AI usage on smartphone dependency, digital amnesia, and related somatic symptoms among nursing students. This can offer insights into how these issues develop and change over time.

## Electronic supplementary material

Below is the link to the electronic supplementary material.

Supplementary Material 1

## Data Availability

The datasets are available from the corresponding author upon reasonable request.

## References

[1] Komalavalli K., et al. (2020). A survey of artificial intelligence in smart phones and its applications among the students of higher education in and around Chennai city. Shanlax Internat Jour Educ. 2020;8(3):89–95.

[2] Dirin A, Alamäki A, Suomala J. Digital Amnesia and Personal Dependency on Smart Devices: A Challenge for Artificial Intelligence. In Ketamo, H. & O’Rourke, P, editors: Proceedings of Fake Intelligence Online Summit 2019, May 7, Pori, Finland. 2019:31–36.

[3] Strielkowski W. COVID-19 pandemic and the digital revolution in academia and higher education. Preprints 2020. 2020;2020040290. 10.20944/preprints202004.0290.

[4] Harkin L, Daria J, Kuss. My smartphone is an extension of myself: A holistic qualitative exploration of the impact of using a smartphone. 2021;10(1):28–38. 10.1037/PPM0000278

[5] Kerimbayev N, Umirzakova Z, Shadiev R et al. (2023). A student-centered approach using modern technologies in distance learning: a systematic literature review. Smart Learn. Environ. 2023;10(61). 10.1186/s40561-023-00280-8

[6] Silva MA, da, Nanamura MHS. The impact of new technologies on medical education. Seven Editora. 2024;284–291. Retrieved from https://sevenpublicacoes.com.br/editora/article/view/4275

[7] Moro C, Phelps C, Redmond P, Stromberga Z. HoloLens and mobile augmented reality in medical and health science education: A randomised controlled trial. Br J Educ Technol. 2021;52:680–94. 10.1111/bjet.13049.

[8] Legner C, Eymann T, Hess T, et al. Digitalization: opportunity and challenge for the business and information systems engineering community. Bus Inf Syst Eng. 2017;59:301–8. 10.1007/s12599-017-0484-2.

[9] Small GW, Lee J, Kaufman A, Jalil J, Siddarth P, Gaddipati H, Moody TD, Bookheimer SY. Brain health consequences of digital technology use. Dialogues Clin Neurosci. 2020;22(2):179–187. 10.31887/DCNS.2020.22.2/gsmall. PMID: 32699518; PMCID: PMC7366948. 10.31887/DCNS.2020.22.2/gsmall PMC7366948 32699518

[10] Song YH, Park TJ. The factors influencing smart devices addiction among korean middle school students in technology-enhanced learning environments. Indian Jour Publ Heal Res Dev. Sep 2018. 2018;9(9) 10.5958/0976-5506.2018.01105.1

[11] Farghaly SM, Dator WLT, Sankarapandian C. The relationship between nursing students’ smart devices addiction and their perception of artificial intelligence. Healthcare. 2023;11(110). 10.3390/healthcare11010110 10.3390/healthcare11010110 PMC9819298 36611570

[12] Ahn JS, Jun HJ, Kim TS. Factors affecting smartphone dependency and digital dementia. J Inform Technol Appl Manage. 2015;22(3):35–54. 10.21219/JITAM.2015.22.3.035.

[13] Lobo LM, Évora YD, Santos AM, Gouveia MT, Andrade EM. Factors associated with smartphone e addiction in nursing students. Texto Contexto Enferm [Internet]. 2022 [cited YEAR MONTH DAY];31:e20210045. Available from: 10.1590/1980-265X-TCE-2021-0045

[14] Lodha P. Digital Amnesia: Are we headed towards another amnesia. Indian J Mental Health. 2019. 10.30877/IJMH.6.1.2019.18-22.

[15] Tanil CT, Yong MH. Mobile phones: The effect of its presence on learning and memory. PLoS One. 2020;15(8):e0219233. 10.1371/journal.pone.0219233. IF: 2.9 Q1. PMID: 32790667IF: 2.9 Q1; PMCID: PMC7425970. 10.1371/journal.pone.0219233 PMC7425970 32790667

[16] Harwood J, Dooley JJ, Scott AJ, Joiner R. Constantly connected—The effects of smart-devices on mental health. Comput Hum Behav. 2014;34:267–72. 10.1016/j.chb.2014.02.006.

[17] Savarimuthu JR, Subramanian K. Associations between digital amnesia, sleep disorders and somatic symptoms among youth. Cognition Brain Behav Interdisciplinary J. 2021;XXVI(2 June):121–35. 10.24193/cbb.2022.26.07.

[18] Theocharis A, Antonopoulos V, Christodoulou NG. Somatic symptoms associated with mental distress during the COVID-19 pandemic: A systematic review. Australas Psychiatry. 2023;31(2):147–156. 10.1177/10398562231156380 10.1177/10398562231156380 PMC9969186 36825513

[19] Zheng F, Duan Y, Li J, et al. Somatic symptoms and their association with anxiety and depression in Chinese patients with cardiac neurosis. Jour Internat Med Res. 2019;47(10):4920–4928. 10.1177/0300060519869711. IF: 1.4 Q4. 10.1177/0300060519869711 PMC6833396 31448660

[20] Priporas C-V, Stylos N, Fotiadis AK. Generation Z consumers’ expectations of interactions in smart retailing: A future agenda. Comput Hum Behav. 2017;77:374–81. 10.1016/j.chb.2017.01.058.

[21] Greenwood C, Quinn M. Digital amnesia and the future tourist. J Tourism Futures. 2017;3(1):73–6. 10.1108/JTF-11-2016-0037.

[22] Cerutti R, Spensieri V, Amendola S, et al. Sleep disturbances partially mediate the association between problematic internet use and somatic symptomatology in adolescence. Curr Psychol. 2021;40:4581–9. 10.1007/s12144-019-00414-7.

[23] Dienlin T, Johannes N. The impact of digital technology use on adolescent well-being. Dialog Clin Neurosci. 2020;22(2):135–42. 10.31887/DCNS.2020.22.2/tdienlin. 10.31887/DCNS.2020.22.2/tdienlin PMC7366938 32699513

[24] Wacks Y, Weinstein AM. Excessive smartphone use is associated with health problems in adolescents and young adults. Front Psychiatry. 2021;12:669042. 10.3389/fpsyt.2021.669042. 34140904 10.3389/fpsyt.2021.669042 PMC8204720

[25] Chatzea VE, Logothetis I, Kalogiannakis M, Rovithis M, Vidakis N. Digital educational tools for undergraduate nursing education: A review of serious games, gamified applications and non-gamified virtual reality simulations/tools for nursing students. Information. 2024;15(7):410. 10.3390/info15070410

[26] Korte M. The impact of the digital revolution on human brain and behavior: Where do we stand? Dialog Clin Neurosci. 2020;22(2):101–11. 10.31887/DCNS.2020.22.2/mkorte. 10.31887/DCNS.2020.22.2/mkorte PMC7366944 32699510

[27] Huang S, Lai X, Ke L, Li Y, Wang H, Zhao X, Wang Y. AI technology panic—is AI dependence bad for mental health? A cross-lagged panel model and the mediating roles of motivations for AI use among adolescents. Psychol Res Behav Manage. 2024;17:1087–102. 10.2147/PRBM.S440889. 10.2147/PRBM.S440889 PMC10944174 38495087

[28] Hassani H, Huang X, Silva E. The Human Digitalization Journey: Technology First at the Expense of Humans? Information. 2021;12(267). 10.3390/info12070267

[29] Winkler A, Jeromin F, Doering BK, Barke A. Problematic smartphone use has detrimental effects on mental health and somatic symptoms in a heterogeneous sample of German adults. Comput Hum Behav. 2020;113:106500. 10.1016/J.CHB.2020.106500.

[30] Zhang D, Tang F, Feng X, Jian-feng L, Lan Y. The impact of mobile phone addiction on daily memory and the mediating role of sleep quality: an empirical study based on Chinese and Filipino college students. Int J Educ Humanit. 2024. 10.54097/m1e3z695.

[31] Nikolic A, Bukurov B, Kocic I, Vukovic M, Ladjevic N, Vrhovac M, Pavlović Z, Grujicic J, Kisic D, Sipetic S. Smartphone addiction, sleep quality, depression, anxiety, and stress among medical students. Front Public Health. 2023;11:1252371. 10.3389/fpubh.2023.1252371. 37744504 10.3389/fpubh.2023.1252371 PMC10512032

[32] Yamane T. Statistics: An Introductory Analysis, 2nd Edition, New York: Harper and Row. 1967.

[33] Kaspersky Lab. Digital Amnesia at work, the risks and rewards of forgetting in business. 2016. http://newsroom.kaspersky.eu/fileadmin/user_upload/de/Downloads/PDFs/Digital_Amnesia_at_work-the_risks_and_rewards_of_forgetting_in_business.pdf

[34] Chóliz M. Mobile-phone addiction in adolescence: The test of mobile phone dependence (TMD). Prog Heal Sci. 2012;2(1):33–44. Retrieved from https://www.proquest.com/scholarly-journals/mobile-phone-addiction-adolescence-test/docview/1335011974/se-2

[35] Gierk B, Kohlmann S, Kroenke K, Spangenberg L, Zenger M, Brähler E, Löwe B. The somatic symptom Scale–8 (SSS-8): A brief measure of somatic symptom burden. JAMA Intern Med. 2014;174(3):399–407. https://jamanetwork.com/journals/jamainternalmedicine/fullarticle/1783305. 24276929 10.1001/jamainternmed.2013.12179

[36] Robert SJ, Kadhiravan S. Prevalence of digital amnesia, somatic symptoms and sleep disorders among youth during COVID-19 pandemic. Heliyon. 2022;8(8):e10026. https://www.cell.com/heliyon/fulltext/S2405-8440(22)01314-7. IF: 3.4 Q1. Epub 2022 Jul 21. PMID: 35880083IF: 3.4 Q1; PMCID: PMC9301932. 10.1016/j.heliyon.2022.e10026 PMC9301932 35880083

[37] Rakesh S, Vibhuti V, Rincy R, Thottiyil EA, Hemkala R, Lata RH. Smartphone dependency and its impact on academics among medical and nursing students: A cross-sectional study. Jour Integ Nur. 2022;4(1):30–35, Jan–Mar 2022.| 10.4103/jin.jin_44_21

[38] Turan N, Durgun H, Kaya H, Aşti T, Yilmaz Y, Gündüz G, Kuvan D, Ertaş G. Relationship between nursing students’ levels of internet addiction, loneliness, and life satisfaction. Perspect Psychiatr Care. 2020;56(3):598–604. 10.1111/PPC.12474. 31970780 10.1111/ppc.12474

[39] Kalal N, Vel NS, Angmo S, Choyal S, Bishnoi S, Dhaka S, Rulaniya S, Banswal S. Smartphone addiction and its impact on quality of sleep and academic performance among nursing students. Institutional based cross-sectional study in Western Rajasthan (India). Investigacion y Educacion en Enfermeria. 2023;41. 10.17533/udea.iee.v41n2e11. PMC10599697 38589329

[40] Kim YH. Effect of smartphone addiction on memory loss in nursing students: Mediating effect of depression. Korean Association For Learner-Centered Curriculum And Instruction; 2023.

[41] George TP, DeCristofaro C. Use of smartphones with undergraduate nursing students. J Nurs Educ. 2016;55(7):411–5. 10.3928/01484834-20160615-11. 27351612 10.3928/01484834-20160615-11

[42] Backåberg S, Rask M, Brunt D, Gummesson C. Impact of musculoskeletal symptoms on general physical activity during nursing education. Nurse Educ Pract. 2014;14(4):385–90. 10.1016/j.nepr.2014.02.003. 24594281 10.1016/j.nepr.2014.02.003

[43] Firmino CI, Sousa LM, Rosa PJ, Marques MF, Simões MC. Musculoskeletal symptoms in nursing students: The role of psychosocial factors. Revista De Enfermagem Referência. 2021;5(5):e20085. 10.12707/RV20085.

[44] Sperling EL, Hulett JM, Sherwin LB, Thompson S, Bettencourt BA. Prevalence, characteristics and measurement of somatic symptoms related to mental health in medical students: A scoping review. Ann Med. 2023;55(2). 10.1080/07853890.2023.2242781. 10.1080/07853890.2023.2242781 PMC10411307 37552776

[45] Toda M, Ezoe E, Mure K, Takeshita. T. Relationship of smartphone dependence to general health status and personality traits among university students.january 2016 open. J Prev Med. 2016;06(10):215–21. 10.4236/ojpm.2016.610020.

[46] Musa N, Mukhtaruddin, Bakkara VF. The Effects of Digital Amnesia on Knowledge Construction and Memory Retention. Khizanah Al-Hikmah: Jurnal Ilmu Perpustakaan, Informasi, Dan Kearsipan. 2023;11(2). Retrieved from https://journal.uin-alauddin.ac.id/index.php/khizanah-al-hikmah/article/view/43959. DOI: 10.24252/kah.v11i2cf1

[47] Bhanderi DJ, Pandya YP, Sharma DB. Smartphone use and its addiction among adolescents in the age group of 16–19 years. Indian J Community Medicine: Official Publication Indian Association Prev Social Med. 2021;46(1):88–92. 10.4103/ijcm.IJCM_263_20. 10.4103/ijcm.IJCM_263_20 PMC8117907 34035584

[48] Atalla ADG, El-Ashry AM, Mohamed S, Mohamed. The moderating role of ethical awareness in the relationship between nurses’ artificial intelligence perceptions, attitudes, and innovative work behavior: a cross-sectional study. BMC Nurs. 2024;23:488. 10.1186/s12912-024-02143-0. 39026317 10.1186/s12912-024-02143-0 PMC11256689

