# Supplementary material for: Smartphone dependency, digital amnesia, and somatic symptoms among nursing students: the challenge of artificial intelligence
Source: BMC Nurs. 2025 May 26;24:599. doi: 10.1186/s12912-025-03228-0 (PMC12107735; doi:10.1186/s12912-025-03228-0)
Supplement: Supplementary file 1 — Supplementary Material 1 [file 12912_2025_3228_MOESM1_ESM.docx]

**Artificial intelligence usage questionnaire**

**Part (I): Personal Characteristics Data**

1. **Gender: 1-** Male () 2- Female ( )
2. **Age**: ------ 18-20Yrs ( ) 21-23Yrs ( ) 24-28Yrs ( ) .
3. **Marital status: 1-** Single ( ) 3- Married ( ) 4- Widowed ( ) 5- Divorced ( )
4. **Residential area** urban( ) rural( )
5. **Name of university**
6. **Educational level:** first( ) second( ) third( ) fourth( ) fifth( ) intern student( )

**Part II; Artificial intelligence usage questionnaire:**

1. **Have you heard of the term artificial intelligence?** Yes ( ) no ( )
2. **Uses of AI for** Education ( ) Entertainment ( ) both ( )
3. **Which AI app or tools do you mostly use?**

ChatGPT ( )

Google Assistant / Google translate / Google lens ( )

Smart camera app (for photo / camera editor) ( )

Grammarly ( )

True caller ( )

Face recognition ( )

Smart scan ( )

GPS ( for traffic patterns, road conditions) ( )

Siri ( )

Share it( )

1. **How often do you use artificial intelligence apps?** Rarely( ) several times a week( ) almost every day ( )
